# Efficacy of Epley's Maneuver in Treating BPPV Patients: A Prospective Observational Study

**DOI:** 10.1155/2015/487160

**Published:** 2015-10-01

**Authors:** Sushil Gaur, Sanjeev Kumar Awasthi, Sunil Kumar Singh Bhadouriya, Rohit Saxena, Vivek Kumar Pathak, Mamta Bisht

**Affiliations:** Department of E.N.T. and Head & Neck Surgery, School of Medical Science & Research, Greater Noida 201306, India

## Abstract

Vertigo and balance disorders are among the most common symptoms encountered in patients who visit ENT outpatient department. This is associated with risk of falling and is compounded in elderly persons with other neurologic deficits and chronic medical problems. BPPV is the most common cause of peripheral vertigo. BPPV is a common vestibular disorder leading to significant morbidity, psychosocial impact, and medical costs. The objective of Epley's maneuver, which is noninvasive, inexpensive, and easily administered, is to move the canaliths out of the canal to the utricle where they no longer affect the canal dynamics. Our study aims to analyze the response to Epley's maneuver in a series of patients with posterior canal BPPV and compares the results with those treated exclusively by medical management alone. Even though many studies have been conducted to prove the efficacy of this maneuver, this study reinforces the validity of Epley's maneuver by comparison with the medical management.

## 1. Introduction

BPPV was first described by Barany in 1921, and he attributed the disorder to otolith disease [1]. The clinical diagnosis of this disorder was not well defined until Dix and Hallpike described the classic positioning which causes a characteristic nystagmus [2]. Benign paroxysmal positioning vertigo is a disorder characterized by brief attacks of vertigo, with associated nystagmus, precipitated by certain changes in head position with respect to gravity [3]. BPPV is the most common cause of vertigo in patients seen by the otolaryngologist. The incidence is difficult to estimate because of the benign, typically self-limited course of the disease. It is thought to range from 10.7 per 100,000 to 17.3 per 100,000 population in Japan [4] and has been reported as 64 per 100,000 in a population study from Minnesota [5]. The mean age at onset is in the fourth and fifth decades, but BPPV also may occur in childhood. Overall, the incidence increases with age. Symptoms occur suddenly and last on the order of seconds but never in excess of a minute. The subjective impression of attack reported by the patient frequently is longer. In most cases of BPPV, no specific etiologic disorder can be identified. The most common known cause was closed head injury, followed by vestibular neuritis. BPPV will eventually develop in nearly 15% of patients suffering from vestibular neuritis. Other cited predisposing events include infections and certain surgical procedures, including stapedectomy and insertion of a cochlear implant [6]. Prolonged bed rest and Meniere's disease [7] also are predisposing factors. Schuknecht observed granular deposits on the cupula of the posterior semicircular canal in temporal bone specimens and proposed the “cupulolithiasis” theory to explain the pathophysiology. This theory provides a basis for understanding the disorder, although more recent work has shown that the disorder is more commonly due to free-floating particles in the semicircular canal (“canalithiasis”), rather than cupulolithiasis. The suggestion that the mechanism of BPPV could result from deflection of the posterior canal cupula by the movement of debris in the posterior canal was revisited by Hall and colleagues [8]. The posterior semicircular canal was affected in the majority of cases of BPPV (93% of cases), with 85% being unilateral and 8% affecting the PSC on both sides. The horizontal semicircular canal was affected in 5% of cases. Involvement of anterior canal is rare. The positioning examination (Dix-Hallpike test) is important for identifying BPPV. A Dix-Hallpike maneuver produces transient vertigo and nystagmus and is diagnostic. The bedside Dix-Hallpike test combined with an appropriate history is key in making the diagnosis [2]. Standard electrooculography and the many videonystagmography devices do not record the torsional eye movements associated with BPPV. It was noted that the disease could be cured by a chemical labyrinthectomy and eighth nerve section. Gacek proposed transection of only the posterior ampullary nerve for relief of BPPV, confirming the posterior canal origin. In most patients, however, Epley's canalith repositioning maneuver is adequate treatment [9], and no surgery is required. First-line therapy for BPPV is organized around repositioning maneuvers. For posterior canal BPPV, the maneuver developed by Epley is particularly effective [10].

## 2. Materials and Method

This prospective observational study was conducted among patients attending the Department of ENT, Sharda Hospital, School of Medical Sciences and Research, Greater Noida, for a period of two years from June 2013 to June 2015. The clinical case patients above 18 years of age with posterior semicircular canal benign positional paroxysmal vertigo were included in this study. Informed written consent was taken from all the patients included in the study. The patients with cervical spondylosis, ongoing CNS disease (stroke or TIA), and cardiovascular disease and pregnant women beyond 24 weeks were excluded from this study. 50 study participants with positive positional test were divided into two groups each consisting of 25 patients. One group of 25 patients who received medical therapy with Epley's maneuver were considered as the cases and the other group of 25 patients who received only medical therapy were considered as the controls. Epley's maneuver will be repeated until symptomatic relief. The results were classified after treatment with and without the Epley maneuver into resolution of vertigo, presence of nonpositional vertigo, partial resolution, and same or worse. The maneuver begins with placement of the head into the Dix-Hallpike position, to evoke vertigo. The posterior canal on the affected side is in the earth vertical plane with the head in this position. After the initial nystagmus subsides, a 180-degree roll of the head to the position in which the offending ear is up is performed. The patient is then brought to the sitting upright position. The maneuver is likely to be successful when nystagmus of the same direction continues to be elicited in each of the new positions. The maneuver is repeated until no nystagmus is elicited. The patients were given concomitantly both the drugs betahistine 16 mg thrice daily and cinnarizine 25 mg twice daily, till the patient had complete resolution of symptoms. We collected baseline information and clinical history and documented the procedures and treatment assigned to the study participants. We followed the patients for one year with review visit at the 1st week, 4 weeks, 3 months, 6 months, and at the end of one year. The followup process was explained to the patients and they were followed up throughout the study period. Response rate was 100%. The identification forms were separated from data collection instruments and kept under lock and key. Preprocedural and postprocedural instructions were given to all the patients who undergo Epley maneuver. The study protocol was approved by Institute Ethical Committee. Chi square test was used to test the significance of association in the observed data.

## 3. Results

The median age of the participants was 55 years and mean age was 53 years with standard deviation of 13 years.  Table 1 compares the age profile of study and control group. The age distribution of patients was comparable between the two groups with no significance in age distribution between the two groups (*p* = 0.500), implying that there is no age related factors in the incidence of adverse events.

Among all participants, 30 participants (60%) were female. Gender ratio was comparable between two groups of patients with no significant difference (Table 2).

The cases and controls were studied for the associated symptoms which may be variable factor among the two groups influencing the results. 19 (38%) patients had associated symptoms of nausea and vomiting. The incidence of the associated symptoms was comparable among the two groups (Table 3).

Hypertension and diabetes were found among 18 (36%) participants (Table 4). This variable may influence the results of the observation of study.

**Figure 1:**
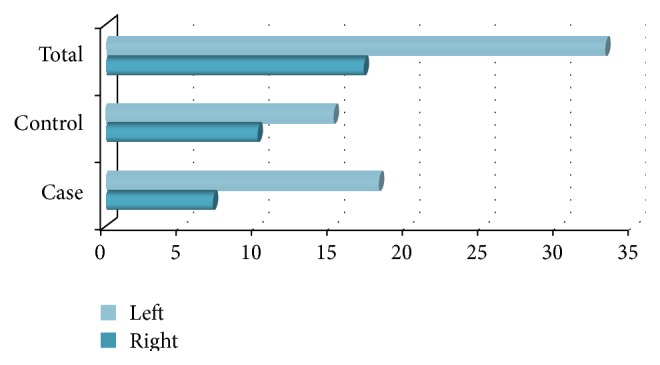
Side of BPPV.

The side of BPPV between the cases and controls was compared. 28% of cases and 40% of controls had right-sided and 72% of cases and 60% of controls had left-sided BPPV (Figure 1).

Among 25 case patients, 18 (72%) recovered from vertigo immediately after the Epley maneuver and 23 (92%) patients recovered from vertigo at first week of followup. The remaining 2 case patients recovered from vertigo during the second and third follow-up visits, whereas, among 25 control patients, 3 (12%) recovered from vertigo at first followup and 19 (76%) participants recovered from the vertigo at third followup. In dose response analysis, control patients needed 2 more visits than case patients; chi square for linear trend was 16.82 and it was significant (*p* value: 0.00004) (Table 5). Case patients were 6 times more likely to recover than control patients (RR: 5.95, 95% CI: 3.85–8.78) and it is statistically significant (*p* < 0.005). The recovery was attributed to Epley maneuver among 67% (95% CI: 43%–81%) of case patients (Table 6).

In regression analysis, preexisting hypertension and diabetes mellitus were confounding the result of Epley maneuver which is evidenced by differing stratum odds ratio (OR: 70.5, Adjusted OR: 55.4, 95% CI: 10.5–457.5, *p* value: <0.05) (Table 7). When controlling the past history, current medications, and associated symptoms, the case patients showed protective Cox proportional hazard ratio of 0.18 (95% CI: 0.06–0.4, *p* value: 0.0007) and it is statistically significant.

## 4. Discussion

BPPV affects all age groups, though it appears to be more common in the elderly. This condition seems to have a predilection for the older population. In our study, the median age of the participants was 55 years and mean age was 53 years with standard deviation of 13 years correlating with the literature [5, 11].

The sex distribution seems to indicate a predilection for women. Among all participants, 30 participants (60%) were female; this is similar to other published reports [5]. Predilection to side was found as left side was affected among 33 (66%) participants. On the other hand some researchers have found that BPPV affects predominantly the right labyrinth [12].

Hypertension and diabetes were found among 18 (36%) participants. Diabetes was found to be unusually prevalent in BPPV patients in a study done by Cohen et al. [13].

In the present study, we found that up to 92% of patients reported benefit after the first follow-up period of one week. In a randomised study, 90% of patients were either improved or cured after a single session with either Semont's or Epley maneuver [14]. Epley himself reported a success rate of more than 90% following a single treatment session. Among 25 case patients, 18 (72%) recovered from vertigo immediately after the Epley maneuver and 23 (92%) patients recovered from vertigo at first week of followup. The remaining 2 case patients recovered from vertigo during second and third follow-up visits, whereas, among 25 control patients, 3 (12%) recovered from vertigo at first followup and 19 (76%) participants recovered from the vertigo at third followup. This clearly indicates the efficacy of Epley maneuver in treatment of BPPV against the medical therapy. In our study labyrinthine sedatives were used in both case and control groups. In the control group of 25 patients, labyrinthine sedatives were given from the time of first visit to the period when patient is symptom-free. Labyrinthine sedatives failed to control the symptoms of BPPV even after a prolonged use, although they may provide minimal relief for some patients.

A review of the literature revealed the extremely good results of the Epley maneuver. In one study, the success rate after 1 week was 63.6%, which increased to 72.7% after 2 weeks [15]. One Brazilian study also revealed similar results [16]. A meta-analysis done by Prim-Espada et al. on the efficacy of Epley's maneuver in benign paroxysmal positional vertigo using a critical review of the medical literature concluded that the patients on whom Epley's maneuver was performed had six and half times more chance of their clinical symptoms improving compared to the control group of patients (OR = 6.52; 95% CI, 4.17–10.20) [17]. The efficacy of Epley's maneuver in the treatment of BPPV was assessed in a study of 62 patients conducted by Khatri et al. Patients were selected based on symptoms of positional vertigo and positive Dix-Hallpike's test. At the end of 1 month patients were assessed subjectively by visual analogue scale (VAS) and objectively by Dix-Hallpike's positional test. On VAS, 85.7% of patients had complete resolution of symptoms of BPPV in both groups. Objectively 88.2% did not have positional nystagmus after 1 month in first group, whereas in the second group 86% had complete response at the end of 1 month of therapy [18].

In a study of four hundred and twelve patients with unilateral benign paroxysmal positional vertigo of the posterior semicircular canal, the patients were treated with the Semont maneuver and if symptoms did not resolve, successive application of three Epley maneuvers plus Brandt-Daroff exercises was given. The study concluded that, in unilateral benign paroxysmal positional vertigo of the posterior semicircular canal, the above treatment protocol cured 98% of patients [19]. In a prospective study liberatory maneuver-betahistine and Brandt-Daroff-betahistine groups did significantly better than liberatory maneuver and Brandt and Daroff groups (*p* < 0.05). This study signifies the added efficiency of betahistine with particle repositioning maneuver in treating BPPV [20]. However there are very few studies which have compared the medical therapy with the particle repositioning maneuver.

## 5. Conclusion 

BPPV is common among the elderly with a sex predilection for women and affecting the left side in majority of patients. Comorbid conditions do have a role in causative factors. In our study, Epley's maneuver was more effective than medicines alone not only in treating the condition but also in preventing the recurrence. This maneuver gave recovery among majority of the case patients during their first visit itself. Those who were treated with medicines alone needed more number of visits than those who were treated with Epley's maneuver and medicines. Epley maneuver can be considered safe and effective procedure to treat benign paroxysmal positional vertigo in majority of patients as a bedside maneuver. After controlling the confounders, Epley maneuver with medicines was found more effective than medicines alone.

## Figures and Tables

**Table 1:** Age profile.

Variables	Category	Case (*n* = 25)		Control (*n* = 25)		Total (*N* = 50)
		*n*	%	*n*	%	*n*
Age	Mean	53 ± 15		53 ± 11		53 ± 13
	<40	6	24	4	16	10
	40–60	11	44	14	56	25
	>60	8	32	7	28	15

**Table 2:** Gender profile.

Variables	Category	Case (*n* = 25)		Control (*n* = 25)		Total (*N* = 50)	
		*n*	%	*n*	%	*n*	%
Gender	Female	15	60	15	60	30	60
	Male	10	40	10	40	20	40

**Table 3:** Associated symptoms.

Variables	Category	Case (*n* = 25)		Control (*n* = 25)		Total (*N* = 50)	
		*n*	%	*n*	%	*n*	%
Associated symptoms	Tinnitus	1	4	2	8	3	6
	Nausea and vomiting	11	44	8	32	19	38
	Nausea, vomiting, and tinnitus	1	4	0	0	1	2

**Table 4:** Presence of associated clinical illnesses.

Variables	Category	Case (*n* = 25)		Control (*n* = 25)		Total (*N* = 50)	
		*n*	%	*n*	%	*n*	%
Systemic diseases	Diabetes	12	48	6	24	18	36
	Hypertension	12	48	6	24	18	36
	CAD and others	3	12	3	12	6	12

**Table 5:** Dose response relationship between Epley's maneuver and controls among BPPV patients.

Level	Followup	Cases	Control	Total	Odds of Exp.	OR	Mantel-Haenszel chi square for linear trend	*p* value
1st course		18	3	21	6	1		
2nd course	1st	5	10	15	0.5	0.08		
3rd course	2nd	1	6	7	0.17	0.03	16.82	0.00004115
4th course	3rd	1	6	7	0.17	0.03		
Total		25	25	50				

**Table 6:** Efficacy of Epley's maneuver based on person time among benign paroxysmal positional vertigo patients.

Point estimates		95% confidence interval		*p* value
Type	Value	Lower	Upper	
Conditional maximum likelihood estimate of RR (CMLE)	3.071	1.69	5.57	0.00018
Rate in the exposed	5.95	3.85	8.78	
Rate in the unexposed	1.93	1.25	2.86	
Rate difference	4.01	1.56	6.46	
Attributable fraction in exposed	67.44%	43.30%	81.30%	
Attributable fraction in population	33.72%	15.80%	51.63%	

**Table 7:** Factors affecting the efficacy of Epley's maneuver among BPPV patients.

Variables	Response	Case	Control	OR		95% CI		*p* value
				Crude	Adjusted	Lower	Upper	
Hypertension	Yes	11	1	70.5	55.4	10.5	457.5	<0.005
Diabetes mellitus	Yes	11	1	70.5	55.4	10.5	457.5	<0.005
Past H/o giddiness	Yes	3	0	70.5	107.04	14.17	2846.17	<0.005
On medication	Yes	1	0	70.5	46.4	8.4	410.07	<0.005

## References

[1] Bárány E. (1920). Diagnose yon krankheitserscheinungen im bereiche des otolithenapparates. *Acta Oto-Laryngologica*.

[2] Dix M. R., Hallpike C. S. (1952). The pathology, symptomatology and diagnosis of certain common disorders of the vestibular system. *Annals of Otology, Rhinology & Laryngology*.

[3] Parnes L. S., Agrawal S. K., Atlas J. (2003). Diagnosis and management of benign paroxysmal positional vertigo (BPPV). *Canadian Medical Association Journal*.

[4] Froehling D. A., Silverstein M. D., Mohr D. N., Beatty C. W., Offord K. P., Ballard D. J. (1991). Benign positional vertigo: incidence and prognosis in a population-based study in Olmsted County, Minnesota. *Mayo Clinic Proceedings*.

[5] Baloh R. W., Honrubia V., Jacobson K. (1987). Benign positional vertigo: clinical and oculographic features in 240 cases. *Neurology*.

[6] Viccaro M., Mancini P., La Gamma R., De Seta E., Covelli E., Filipo R. (2007). Positional vertigo and cochlear implantation. *Otology & Neurotology*.

[7] Gross E. M., Ress B. D., Viirre E. S., Nelson J. R., Harris J. P. (2000). Intractable benign paroxysmal positional vertigo in patients with Meniere's disease. *Laryngoscope*.

[8] Hall S. F., Ruby R. R. F., McClure J. A. (1979). The mechanics of benign paroxysmal vertigo. *Journal of Otolaryngology*.

[9] Epley J. M. (1992). The canalith repositioning procedure: for treatment of benign paroxysmal positional vertigo. *Journal of Otolaryngology—Head & Neck Surgery*.

[10] Wolf M., Hertanu T., Novikov I., Kronenberg J. (1999). Epley's manoeuvre for benign paroxysmal positional vertigo: a prospective study. *Clinical Otolaryngology and Allied Sciences*.

[11] Marciano E., Marcelli V. (2002). Postural restrictions in labyrintholithiasis. *European Archives of Oto-Rhino-Laryngology*.

[12] Von Brevern M., Seelig T., Neuhauser H., Lempert T. (2004). Benign paroxysmal positional vertigo predominantly affects the right labyrinth. *Journal of Neurology, Neurosurgery & Psychiatry*.

[13] Cohen H. S., Kimball K. T., Stewart M. G. (2004). Benign paroxysmal positional vertigo and comorbid conditions. *ORL*.

[14] Herdman S. J., Tusa R. J., Zee D. S., Proctor L. R., Mattox D. E. (1993). Single treatment approaches to benign paroxysmal positional vertigo. *Archives of Otolaryngology: Head and Neck Surgery*.

[15] Waleem S. S. U., Malik S. M., Ullah S., ul Hassan Z. (2008). Office management of benign paroxysmal positional vertigo with Epley's maneuver. *Journal of Ayub Medical College, Abbottabad*.

[16] Teixeira L. J., Machado J. N. P. (2006). Manoeuvres for the treatment of benign positional paroxysmal vertigo: a systematic review. *Brazilian Journal of Otorhinolaryngology*.

[17] Prim-Espada M. P., De Diego-Sastre J. I., Pérez-Fernández E. (2010). Meta-analysis on the efficacy of Epley's manoeuvre in benign paroxysmal positional vertigo. *Neurologia*.

[18] Khatri M., Raizada R. M., Puttewar M. P. (2005). Epley's canalith-repositioning manoeuvre for benign paroxysmal positional vertigo. *Indian Journal of Otolaryngology and Head and Neck Surgery*.

[19] Soto-Varela A., Rossi-Izquierdo M., Martínez-Capoccioni G., Labella-Caballero T., Santos-Pérez S. (2012). Benign paroxysmal positional vertigo of the posterior semicircular canal: efficacy of Santiago treatment protocol, long-term follow up and analysis of recurrence. *Journal of Laryngology and Otology*.

[20] Cavaliere M., Mottola G., Iemma M. (2005). Benign paroxysmal positional vertigo: a study of two manoeuvres with and without betahistine. *Acta Otorhinolaryngologica Italica*.

